# Visual Strain Sensors Based on Fabry–Perot Structures for Structural Integrity Monitoring

**DOI:** 10.3390/s24113676

**Published:** 2024-06-06

**Authors:** Qingyuan Chen, Furong Liu, Guofeng Xu, Boshuo Yin, Ming Liu, Yifei Xiong, Feiying Wang

**Affiliations:** 1 Key Laboratory of Trans-Scale Laser Manufacturing, Beijing University of Technology, Ministry of Education, Beijing 100124, China; qychen@emails.bjut.edu.cn (Q.C.);; 2 Beijing Engineering Research Center of Laser Technology, Beijing University of Technology, Beijing 100124, China; 3 Faculty of Materials and Manufacturing, Beijing University of Technology, Beijing 100124, China

**Keywords:** structural color, flexible, Fabry–Perot cavity, color reflector, strain sensor

## Abstract

Strain sensors that can rapidly and efficiently detect strain distribution and magnitude are crucial for structural health monitoring and human–computer interactions. However, traditional electrical and optical strain sensors make access to structural health information challenging because data conversion is required, and they have intricate, delicate designs. Drawing inspiration from the moisture-responsive coloration of beetle wing sheaths, we propose using Ecoflex as a flexible substrate. This substrate is coated with a Fabry–Perot (F–P) optical structure, comprising a “reflective layer/stretchable interference cavity/reflective layer”, creating a dynamic color-changing visual strain sensor. Upon the application of external stress, the flexible interference chamber of the sensor stretches and contracts, prompting a blue-shift in the structural reflection curve and displaying varying colors that correlate with the applied strain. The innovative flexible sensor can be attached to complex-shaped components, enabling the visual detection of structural integrity. This biomimetic visual strain sensor holds significant promise for real-time structural health monitoring applications.

## 1. Introduction

Detection of material deformation is key to advanced sensing technologies that provide intelligent monitoring systems. These systems are pivotal not only in human healthcare research [1,2] but also in assessing the structural health of buildings [3,4]. Every year, engineered structures such as bridges and skyscrapers succumb to failure, leading to casualties, death, and significant property damage [5,6]. In response, a variety of structural safety inspection systems have been developed, including wireless sensors [7] and radio frequency identification (RFID) devices [8], to evaluate the integrity of these structures. However, the complexity of these systems necessitates specialized training for installation and operation, resulting in high costs.

Before catastrophic failure, building structures typically show early warning signs, such as cracking and deformation. Optical strain sensors provide a direct method to detect external stress stimuli compared to their electrical counterparts [9,10,11,12,13,14]. In the field of optical strain sensing, optical fiber-based strain sensors were first proposed in the 1980s [15,16], which can sense by detecting optical signals such as optical intensity and phase before and after the strain. However, fiber-optic strain sensors are complicated to prepare and require interfacing with external devices to transmit optical signals, making it impossible to visually observe the strain state. Devices that alter their inherent color in response to external strain stimuli have broad applicability, ranging from human–computer interactions [1] to structural health monitoring [3,4]. By attaching an optical sensor to the surface of a structure, the extent of damage can be gauged through the color change of the sensor, thereby enabling the prediction and prevention of potential structural failure.

Materials and designs that undergo color changes induced by applied force have been the subject of extensive research. Particularly, photonic-crystal-based strain sensors, which directly detect strain via structural color changes, have garnered significant interest [17]. The correlation between color change and strain allows these photonic crystals (PCs) to map strain distribution visually. Nonetheless, the fabrication of PCs involves the sequential layering of different materials, which can lead to structural periodicity disruptions as a result of mismatches in elastic moduli when subjected to external stresses. This disruption often results in decreased color intensity or saturation [17]. Moreover, the production of spatially uniform PCs is a labor-intensive and costly process, leading to low device yields and inconsistent quality. Consequently, there is a pressing demand for a visually based, flexible strain sensor design that offers precise sensing capabilities and is simple to manufacture.

**Figure 1:**
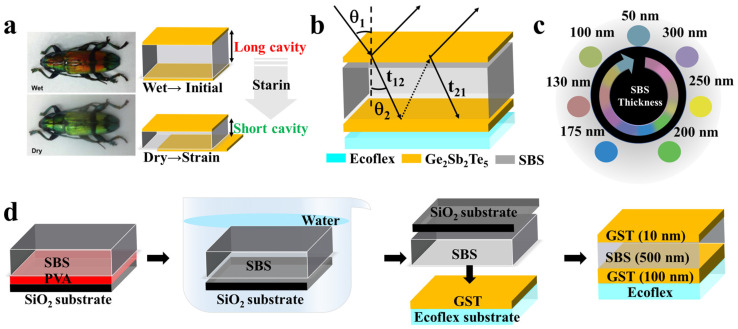
Fabrication of F–P-based flexible visual strain sensor. (**a**) Principle of the phenomenon of hygroscopic coloration in the longhorn beetle; (**b**) Interference effects of incident light in the F-P structure; (**c**) Demonstration of different colors produced by SBS thickness variation; (**d**) Preparation process of F-P-based visualized strain sensors.

Principles of natural selection and competition have driven the design of unique architectures. Structural colors, which emerge from the physical interaction of visible light with optical interference structures rather than pigments or dyes, have garnered significant interest. These colors are prevalent in nature, visible in butterfly wings, fish scales, and beetle shells, and have inspired advancements in artificial colors and optical sensing devices. The dynamic nature of biological structural colors, which adapt in response to environmental changes, is particularly intriguing. For example, sheath wings of the longhorn beetle exhibit a green hue in dry conditions and transition to red when exposed to moisture (Figure 1a). This phenomenon is attributed to a Fabry–Perot (F–P) interference structure on the wing surface, which comprises a “reflective layer-interference cavity-reflective layer”. The interference cavity, which can absorb ambient moisture, expands and alters the interference band of incident light, yielding controllable reflections and a red-shift in the reflectance curve of the F–P structure, resulting in moisture-induced coloration. As depicted in Figure 1b, incident light on the F–P structure undergoes reflection and refraction at the upper interface, and the refracted light traverses the film and undergoes partial reflection and refraction at the lower interface. This cyclical process between the film interfaces results in light waves with varying propagation paths and phase differences, leading to phase-length interference or phase-cancellation interference. These interactions dictate the reflected light components from the structure, thereby generating a distinct structural color. Figure 1c shows the range of structural colors produced by this interference structure at varying interference cavity lengths.

The F–P structure has received a lot of attention due to its advantages of a simple process and wide range of applications. Li et al. [18] proposed a symmetric F–P structure consisting of Ag/SiO_2_/Ag cavities with rich colors obtained by varying the thickness of SiO_2_. Yang et al. [19] obtained three primary RGB (red, green, and blue) reflective colors by fabricating an asymmetric F–P structure consisting of an Si dielectric film sandwiched by a thin chromium (Cr) layer and a thick silver (Ag) layer. However, it is worth noting in these studies of F–P structures generating structural color that once the structure preparation is completed, the structural color cannot be changed and regulated, and can only be used as a static color display image. In our previous study [20,21], the F–P structure was able to produce significant color changes by introducing a Ge_2_Sb_2_Te_5_ (GST) phase-change material as a reflective layer capable of generating reversible switching between crystalline and amorphous states under heating and laser stimulation. However, compared with the interference cavity, the reflective layer material has limited modulation of the reflective state of the structure to further utilize the application potential of the F–P structure. Drawing inspiration from the sheath wings of beetles and leveraging the F–P structure, we have designed an economical, recyclable visual strain sensor that operates independently of an external power supply and readout system. By configuring the interference cavity with a stretchable and flexible material that contracts under external strain and pairing it with a flexible substrate, we have created a sensor that visually indicates structural stress and have demonstrated its feasibility in monitoring structural crack initiation and extension.

## 2. Materials and Methods

### 2.1. Experimental

To construct a stretchable F–P structure, as shown in Figure 1d, we utilized a polystyrene–polybutadiene–polystyrene (SBS) triblock copolymer film as the interference cavity material. SBS, a thermoplastic elastomer, has remarkable mechanical strength (tensile strength > 10 MPa) and extensibility (>500% strain) [22,23], with a high viscosity and a Poisson’s ratio (*ν*) of approximately 0.5 [24]. Consequently, when the sensor is elongated, lateral compression of the spacer layer is directly proportional to ν, resulting in a thickness alteration of the spacer layer for tensile strain ε expressed as Δd=dνε.

Precise control over F–P layer thickness is imperative to achieve the targeted specific color. The fabrication process, shown in Figure 1d, started with a hydrophilic treatment of a glass substrate. Subsequently, a polyvinyl alcohol (PVA) solution was spin-coated onto the glass substrate at 3000 rpm for 1 min and cured at 100 °C for 1 h. The SBS solution was then spin-coated on the PVA layer and dried at 60 °C for 30 min. The glass substrate, coated with PVA and SBS, was immersed in water to selectively dissolve the PVA. By exerting pressure on the reverse side of the glass substrate, the residual SBS layer was transferred onto an Ecoflex flexible substrate pre-coated with a GST film, followed by drying to enhance the uniformity of the SBS layer. The “GST-SBS” films were sequentially layered above the SBS layer. The Ecoflex flexible substrate was synthesized by blending Ecoflex silicone (Smooth-On Ecoflex 00-30, Macungie, PA, USA) gels A and B in a 1:1 mass ratio and allowing it to cure for 24 h. The GST film was deposited onto wafers via magnetron sputtering in a vacuum chamber at a pressure of 1 × 1^−5^ Pa.

### 2.2. Simulation and Methods

Reflectance spectra of the structures were acquired using a Shimadzu UV-3600 spectrophotometer (Sydney, Australia) calibrated against a barium sulfate standard. Sample imaging was conducted under natural lighting with a digital camera without polarizers. Using finite element theory and COMSOL Multiphysics 6.1, a two-dimensional model of the F–P structure was constructed to simulate reflectance and electric field distributions. The methodology for these determinations has been previously described [21]. The simulated reflectance spectra were projected onto a Commission Internationale de l’Éclairage (CIE) 1931 chromaticity diagram, where the corresponding colors were rendered through a reflectance color-transformation formula.

## 3. Results and Discussion

### 3.1. Characteristics of the Visual Strain Sensor

**Figure 2:**
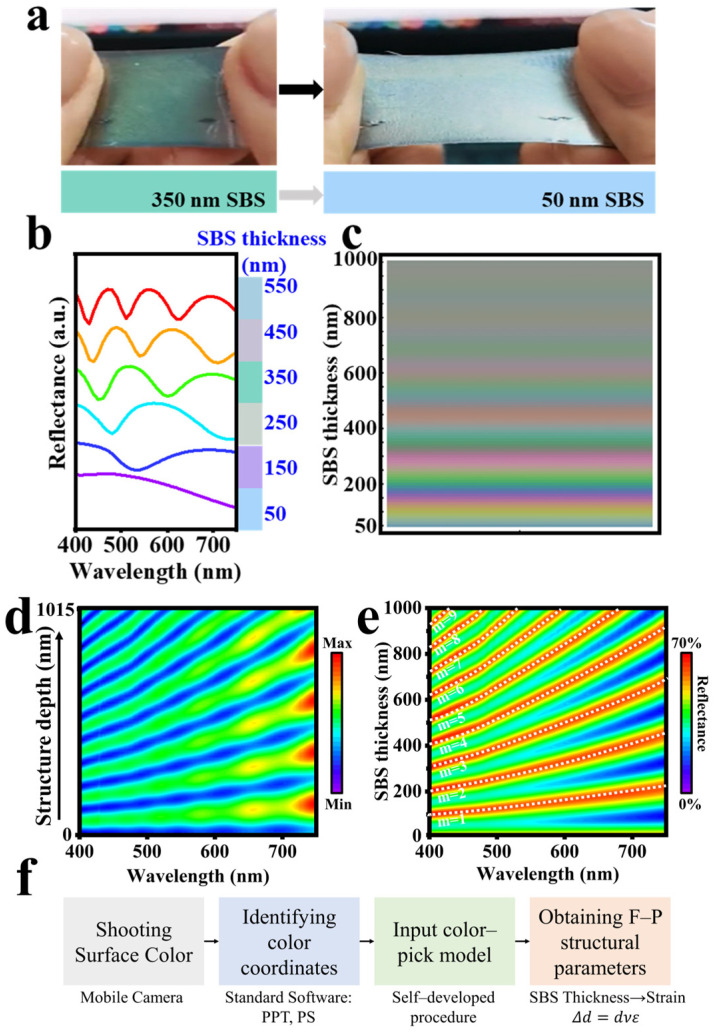
(**a**) Color transition upon strain application; (**b**) reflection curves and colors at varying SBS thicknesses; (**c**) color patterns for different SBS thicknesses; (**d**) electric field distribution with a 1000 nm indium tin oxide layer; and (**e**) reflectance changes with wavelength and SBS thickness. All structures have a top GST layer of 5 nm and a bottom layer of 10 nm; (**f**) flowchart of color–structural parameters’ degree of strain conversion.

As shown in Figure 2a, we fabricated a flexible visual sensing structure composed of “Ecoflex/GST/SBS/GST”. Upon stretching, the sensor color changed from green to light blue. Figure 2b shows a progressive blue-shift in the reflection curve associated with a reduction in SBS interference cavity length, accompanied by a decrease in reflection peaks. This blue-shift is attributed to an increased interference path, resulting in increased resonant absorption within the high-frequency band. This result is consistent with the Fresnel principle, where the resonant peak wavelength (λ) is determined by Equation (1).

(1) $$\lambda =2d\cdot \sqrt{n^{2}-sin^{2}\theta }$$

Here, *d* denotes the interference cavity thickness, *n* is the refractive index of the cavity material, and *θ* is the angle of incident light. According to this equation, *λ* increases with an increase in SBS thickness (*d*). The initial thickness of the SBS interference cavity, as shown in Figure 2a, was 350 nm, which, upon stretching, reduced to 50 nm. Figure 2c integrates the color spectrum resulting from SBS thickness variation, revealing a gradual blue-shift as the thickness decreased, with a value of approximately 150, indicating enhanced color brightness.

To understand the effect of the SBS cavity on the structural interference effect and the origin of the structural color, we calculated the electric field intensity distribution from 400 to 750 nm for an SBS cavity length of 1000 nm, as shown in Figure 2d. The electric field mainly concentrated within the interference cavity, resulting in multilayer localization or multilevel resonance interference. Figure 2e also demonstrates simulated reflectivity variation correlating with SBS cavity length and wavelength. The analysis reveals nine distinct maximum reflectivity bars (white dashed lines) when the SBS cavity length reached 1000 nm, corresponding to the electric field distribution and underscoring the key role of interferometric cavity length on the interreference effect of the F–P structure. To connect the color with strains quantitatively, the surface color was firstly captured by a mobile camera, and then color coordinates were identified with such standard software as PowerPoint, Photoshop, etc. Subsequently, these coordinate data were input into a self-developed color-pick module to determine the structural parameters by extrapolating the color disk shown in Figure 2c. The relationship between color and F–P structural parameters can be also obtained by following the flow chart as given in Figure 2f.

### 3.2. Effects of Upper and Lower Double-GST Layers

**Figure 3:**
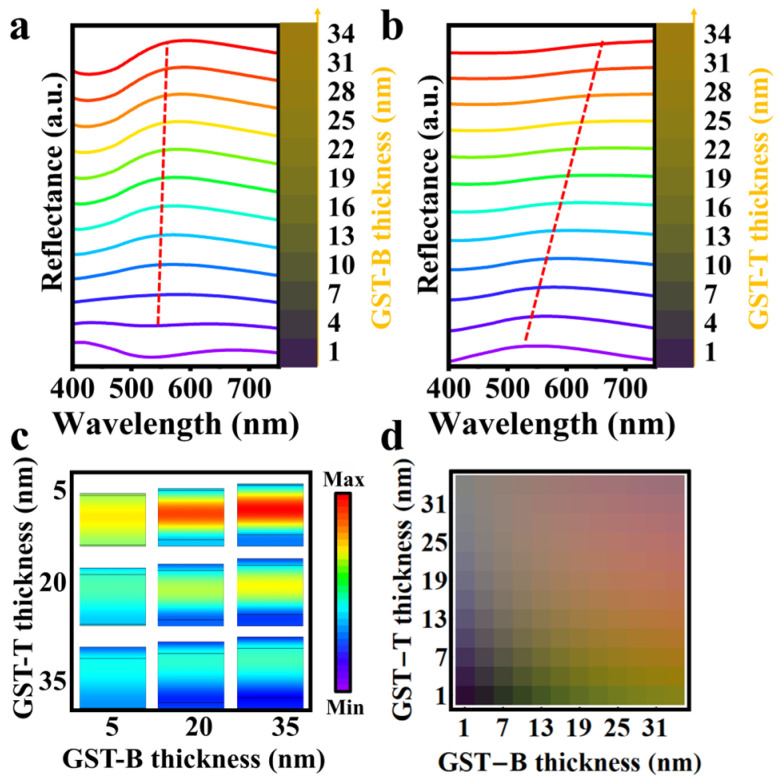
(**a**) reflectance spectra and colors with varied GST-B thicknesses; (**b**) reflectance spectra and colors with varied GST-T thicknesses; (**c**) electric field at peak wavelengths for different GST-B and GST-T thicknesses; and (**d**) colors for various SBS thicknesses.

To elucidate the influence of the lower (GST-B) and upper (GST-T) GST reflective layers on structural interference, we examined the reflectance spectra and structural colors of the GST-B and GST-T layers with different thicknesses, as shown in Figure 3a and Figure 3b, respectively. We observed that increasing the thickness of both GST-T and GST-B layers (ranging from 1 to 34 nm) induces a red-shift in the reflectance curves, alongside an enhancement in the color saturation of the structure. Notably, increasing GST-B layer thickness amplified the reflection peaks, whereas the full width at half maximum (FWHM) of these peaks narrowed. This effect arises because a thicker bottom reflective layer impedes the transmission of incident light out of the structure, thereby extending the interference loop and enhancing the F–P resonance effect, which results in more pronounced selective absorption at the interface.

Although the increase in GST-T layer thickness has a small effect on the shape of the reflection peaks, it significantly influences their position. This is because a thicker GST-T layer restricts the entry of incident light into the structure for interference, and most light is refracted back into the air. However, a greater GST-T thickness introduces additional interfacial phase differences to the incident light, thus resulting in a greater effect on the reflection peak positions of the structure. Consequently, the thicknesses of the GST-T and GST-B layers are pivotal in confining the amount of incident light within the interference layer and determining the strength of F–P resonance.

To corroborate these findings, we calculated the electric field distributions at the reflection peak wavelength for different thicknesses of the GST-T and GST-B structures, as shown in Figure 3c. The results indicate that at a constant GST-T thickness, the electric field strength within the structure progressively increases as the GST-B thickness increases. Conversely, with a fixed GST-B thickness, the electric field strength within the structure gradually decreases with an increase in GST-T thickness. A higher electric field intensity signifies a more robust F–P resonance effect within the structure and a greater saturation of the structural color.

This analysis demonstrates that in the F–P structure, the GST-B layer functions as a barrier to incident light, whereas the GST-T layer facilitates the penetration and reflection of this light. The role of the upper and lower reflective layers is to enable the incident light to undergo multiple interference resonances within the F–P cavity, leading to phase accumulation at the interface and selective reflection of certain visible light wavelengths, thereby yielding highly saturated structural colors. Figure 3d shows the color palette generated by the structure with a set SBS cavity thickness, which was modulated by changing the thicknesses of the GST-T and GST-B layers. As the thicknesses of these layers increase, the color transitions from yellow–green to orange–red, indicating that the reflection curve shifts toward the longer-wavelength region. This suggests that modulating the thicknesses of the upper and lower double-GST layers can also result in significant changes in structural color, thereby enriching the capability of the F–P structure to produce a diverse range of colors.

### 3.3. Visualizing the Color Representation of Strain Sensors

**Figure 4:**
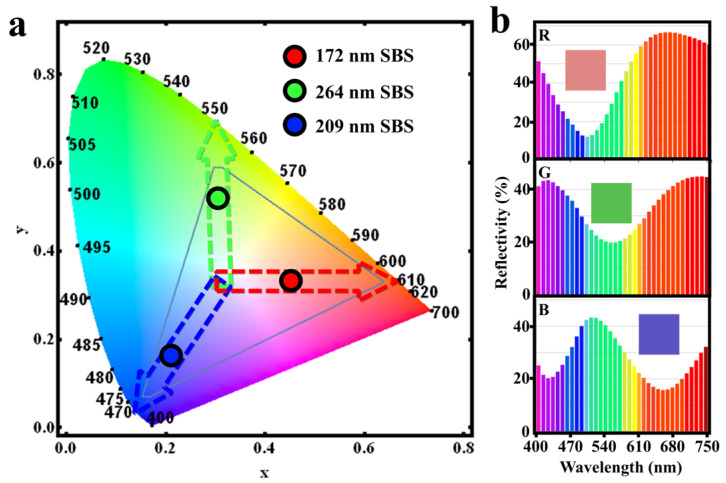
(**a**) CIE-1931 coordinates for RGB with an upper GST layer of 5 nm and a lower layer of 10 nm, and (**b**) reflectance spectra and color blocks for RGB. SBS thicknesses of the F–P structures corresponding to the R, G, and B colors exhibited in Figure 4a and Figure 4b are 172 nm, 264 nm, and 209 nm, respectively.

To assess the color performance of the F–P structure, we examined the red/green/blue (RGB) colors produced. The RGB color coordinates on the CIE-1931 chromaticity diagram are shown in Figure 4a. The position of the RGB trichromatic colors to the edge of the sRGB color space (the triangular area in the figure) suggests that the F–P structure can achieve structural colors with high saturation levels. Figure 4b displays the reflection curves and colors of the RGB trichromes, indicating that variations in the thickness of SBS within the structure cause shifts in the position of the reflection peaks, thereby confirming the critical role of SBS in color modulation.

**Figure 5:**
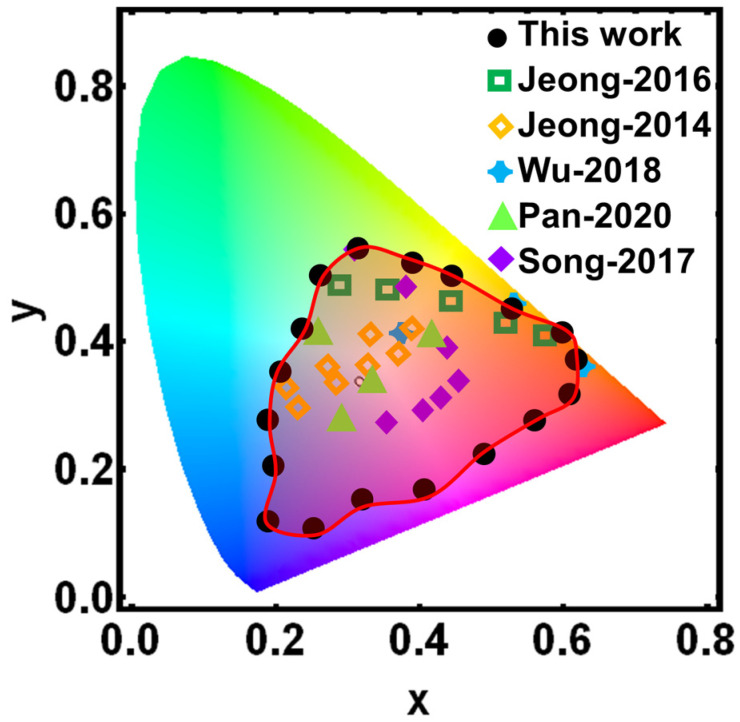
Comparison of strain color coordinate distributions of visual strain sensors designed in this and other studies in CIE-1931 color diagrams [25,26,27,28,29].

To further validate the results of this study, we compared the obtained color gamut with the results of other visual strain sensors in the literature (fluorescent molecular-based [25,26,27], photonic crystal-based [28], metastructure-based sensors [29], etc.), and demonstrated the color changes produced by the sensors under strain in the CIE-1931 diagram as shown in Figure 5. The color gamut in this paper was obtained by performing a thickness scan (0~1000 nm) of the interference cavity of the structure. In previous reports, fluorescent molecular-based, photonic crystal-based, or metastructure-based visualized strain sensors are complicated and expensive to fabricate, and the color change due to strain is not obvious, which does not satisfy the demand for strain degree visualization. In this study, a flexible stretchable SBS material is used as the interferometric cavity material of the F–P structure, which enables the interferometric cavity to be shortened by stretching under force, resulting in a large shift in the reflection peak position, making it possible to produce a significant color change in the structure. As shown in Figure 5, the color gamut (black dots) obtained based on this structure shows a larger color gamut than previous reports [25,26,27,28,29], which results in a more significant color change and a higher degree of strain visualization under external strain. As a result, strain sensors based on an F–P flexible cavity have good application value and commercial potential.

**Figure 6:**
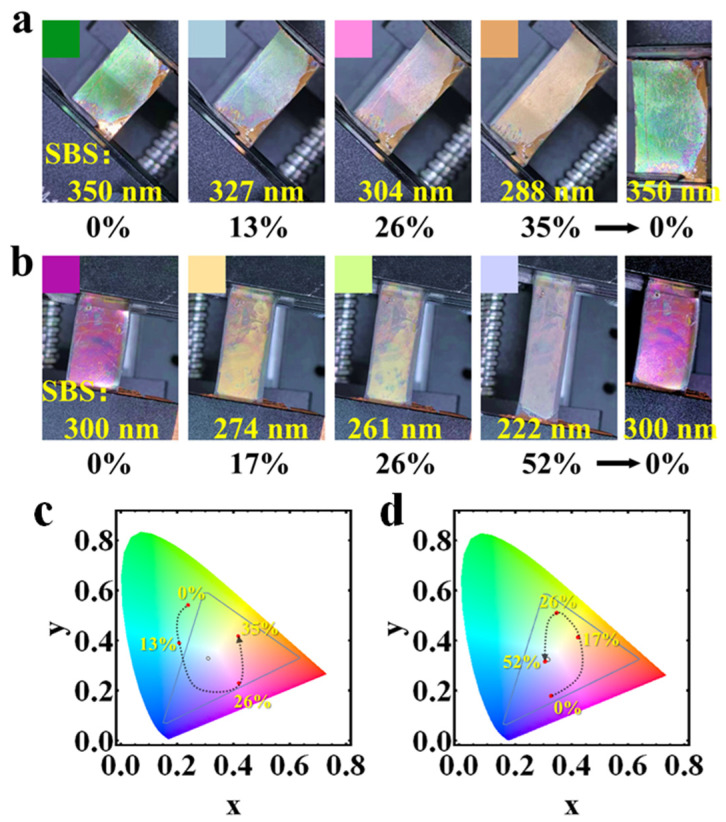
(**a**) sensor with a 300 nm SBS cavity under strain; (**b**) sensor with a 180 nm SBS cavity under strain; (**c**) CIE-1931 coordinates for a 300 nm SBS cavity at different strains; and (**d**) CIE-1931 coordinates for a 180 nm SBS cavity at different strains.

Given that most failures in engineering structures are caused by localized damage, the early detection of localized deformations, such as cracking, disintegration, and non-uniform stress–strain fields, is vital. Figure 6 presents the color changes exhibited by SBS visualization sensors at different strains, corresponding to various SBS thicknesses. Figure 6a depicts the force–light response of the sensor with an initial SBS thickness of 350 nm, where the color transitions from green to cyan blue, fuchsia, and orange, accompanied by a red-shift in hue as strain increases. Concurrently, SBS thickness decreases from 350 to 288 nm under stress. Similarly, the sensor with a 300 nm SBS thickness, shown in Figure 6b, also demonstrates a blue-shift in color (from fuchsia to yellow, then green, and finally blue–violet) as strain is applied. As discussed in Section 3.1, the length of the interferometric cavity significantly influences the wavelength of the reflection peaks; a reduction in cavity length results in a blue-shift of the peaks, which correlates with increased strain. The colors demonstrated in Figure 6a,b are not uniform. That is possibly because of two reasons: (1) some microcracks are created during elongation of the sensor, which affect local optical interference; (2) the spin-coating is not so uniform at the nanoscale because of the limitation of experimental conditions. Figure 6c,d show the CIE coordinate shifts for the two sensing structures with different SBS thicknesses at various strains. With an increase in strain, the color coordinates of the visualized structures move clockwise around the central white point on the diagram. Moreover, the greater the distance of the color coordinate points from the central white point, the higher the color saturation. Comparing the two structures, the structure having a 350 nm SBS cavity exhibits higher color saturation than the structure with a 300 nm SBS cavity. This is attributed to the fact that a longer interferometric cavity length amplifies the F–P interference effect, leading to more intense reflection peaks and increased color saturation. This observation underscores the potential of visual strain sensors in detecting forces on transversely stretched objects. In addition, the final colors of the strain sensor after unloading are also shown in Figure 6a,b, and the same color as its original non-tensile state was found, inferring a good recovery ability of the sensor. In order to obtain the structural change parameters of the sensor during real-time strain, a standard color disk was made in advance similar to the procedure as in Figure 2c,f.

### 3.4. Demonstration of a Structural Health Monitoring Application of Visual Strain Sensors

**Figure 7:**
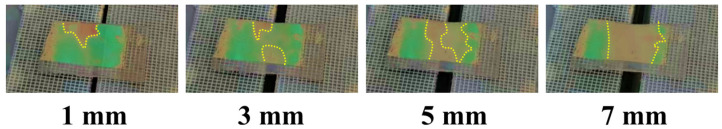
Color distribution in simulated cracks: visual strain sensor color variations for simulated cracks.

Crack initiation and propagation present significant challenges in structural health research. Structural cracks typically result from stresses exceeding the limits of a material, signaling the onset of fracture or damage. Therefore, an intuitive method of monitoring structural health is necessary to monitor the problem of crack initiation and extension. As shown in Figure 7, to simulate cracks in concrete, metal, and other rigid materials, a visual strain sensing structure was affixed to the surface of two flat plates. These plates were separated by a sliding track to simulate “cracks”. The sensor undergoes color transitions from green to red around the gap as a result of stress concentration caused by the separation. As the gap widens, the color distribution of the sensor becomes increasingly uneven, displaying multiple irregular heterochromatic regions. This unevenness arises because the strain in the sensing structure is localized near the gap, resulting in localized color changes. Notably, the heterochromatic regions in the sensing structure exceed the width of the actual slit, enhancing the visibility of crack progression. This demonstration confirms the potential of visual strain sensors based on F–P structures for visualizing localized stresses and monitoring the onset and growth of cracks. To sum up, our visual sensor can give a detailed prediction for damage evolution based on the variation of local colors as shown in Figure 7. A pixel-based design can be developed to show local variations between the initiation and propagation of cracks and colors in the future, which can provide real-time information to alert the initiation of cracks and prevent cracking in advance. 

## 4. Conclusions

We designed a vibrant visual strain sensor by integrating a Fabry–Perot (F–P) interference structure onto a flexible substrate with a stretchable cavity material. The cavity material, SBS, has a Poisson’s ratio (*ν*) of approximately 0.5, meaning the transverse shrinkage of the spacer layer is proportional to *ν* when the sensor is stretched. This characteristic allows the sensor to work in tandem with the F–P structure to achieve pronounced color changes through optical interference. Compared with previous visual strain sensors, the sensor designed in this study further enhances the color gamut area, making the color change by strain more significant. The factors influencing sensor performance and its color change capabilities were thoroughly explored, both theoretically and experimentally. The potential applications of this sensor for strain degree visualization and identification of crack origin/extension were demonstrated. 

## Data Availability

The data presented in this study are available on request from the corresponding author.
